# Visual inspection of root patterns and radiographic estimation of its canal configurations by confirmation using sectioning method. An ex vivo study on maxillary first premolar teeth

**DOI:** 10.1186/s12903-022-02198-y

**Published:** 2022-05-06

**Authors:** Bestoon Mohammed Faraj, Mewan Salahaldeen Abdulrahman, Tavga Mustafa Faris

**Affiliations:** Conservative Department, College of Dentistry, University of Sulaimani, Madame Mitterand Street 30, Sulaimani, Kurdistan Region 46001 Iraq

**Keywords:** Endodontics, Ethnic groups, Geographic locations, Diagnostic imaging conditions, Anatomical pathological

## Abstract

**Background:**

A thorough understanding of the original root and canal anatomy is a critical technical prerequisite for performing cleaning and shaping treatments. Therefore, this research aimed to characterize maxillary first premolar teeth' root morphology and canal architecture.

**Methods:**

One hundred forty-two extracted human adult maxillary first premolar teeth have been retrieved. The extracted teeth were thoroughly cleaned and irrigated to eliminate any remaining debris or blood. They were then preserved in formalin solution until they were eligible for screening. To begin, a visual examination was employed to ascertain the number of roots and their geometry in each sample. Then, utilizing digital radiography in two plains, mesiodistal and distomesial, to further determine those parameters. Finally, a sectioning technique had been used to have the samples cut mesiodistally into slices to validate the internal root canal architecture and identify the number of root canals and their varieties in accordance with Vertucci's categorization system. The canal layout, the pulp chamber, and the root canals were all highlighted (marked) using a fine tip marker in a permanent orange hue to make the canal features more accessible and accurate to visualize. All of the processes were conducted by two highly qualified dentists. The sample size was estimated statistically using the Sealed Envelope program, and the percentage of each configuration was derived in proportion to the overall sample size in order to establish the percentage of each type in each configuration.

**Results:**

From the 142 teeth examined, 42 (29.57%) had one root, 97 (68.31%) had two roots, and only three premolars (2.12%) had three roots. Concerning canal configurations, 100 teeth (70.43%) had type (IV) canal configuration, followed by 37 teeth (26.05%) had types (V), three teeth (2.12%) had type (VIII), and one tooth (0.70%) for each of type (I) and type (II).

**Conclusions:**

The anatomical pattern of inspected maxillary first premolars are mainly two rooted and predominantly have a type (IV) canal morphology.

## Background

The precise orientation about the anatomy of root canals may be a leading cause of treatment failure in endodontics [1]. Hence, a profound and correct experience about the regular and variable root canal system anatomy is crucial for successful endodontic therapy [2]. Several studies were performed to explore maxillary first premolar teeth' root and canal morphology; those studies showed significant alterations according to ancestry and geographic origins [3–6]. Unfortunately, root canals are left untreated when the dentist fails to locate them, particularly in teeth with additional root canals [7].

When one reviews the literature, it becomes apparent that there is a divergence of opinion concerning the root and canal morphology of the first maxillary premolar [8]. Accurate knowledge of the root canal morphology and its anatomical variations is mandatory for successful root canal therapy [9]. Sufficient sample size is essential in studies evaluating root-canal anatomy so that the results are descriptive of the general population [10]. Researchers have documented studies on the number of canals, incidence of multiple roots, complex canal morphology by using different study types like root canal clearing technique, radiographs of root canal treated teeth, in-vitro radiography, sectioning, and analysis of extracted teeth [11]. Several factors that contribute to the dissimilarities observed in anatomic studies in previously documented literature are perhaps due to differences in ethnicity, gender, age, unintentional bias in case of selection, as well as due to in vitro or in vivo study designs [12, 13].

Clinically, before endodontic therapy is performed, it is crucial to identify the root-canal anatomy, the number of roots and canals of the tooth prevalent in a population to reduce errors, to achieve complete debridement and obturation of the canal space during endodontic treatment [14]. However, a deficiency exists in baseline data about the anatomy of maxillary first premolars specific to the Iraqi population. Therefore, we decided to evaluate root and canal morphology in maxillary first premolars from the subpopulation of the Iraqi Kurdistan Region.

## Methods

The Dentistry Ethics Committee approved the present study at the University of Sulaimani (NO.: 2020/433). The applied experimental methods have followed the CRIS guidelines described in the 2014 concept note [15]. The sample size calculation was done using the equation below,

$${\text{n}} = {\text{Z}}\alpha {2}*{\text{P }}\left( {{1} - {\text{P}}} \right)/{\text{d}}\,{2}$$

The sample size is calculated using the aforementioned sample size and taking into account that z = 1.96 and the proportion of single root canals = 10% (ref), with a difference of 0.05 and a power of 80%.;

$$\begin{aligned} {\text{n}} & = \left( {{1}.{96}} \right){2}*0.{1}0 \, \left( {{1}{-}0.{1}0} \right)/\left( {0.0{5}} \right){2} \\ {\text{n}} & = 136 \\ \end{aligned}$$

Then each specimen was examined radiographically by a periapical radiographs. (mesiodistal and distomesial plains) which has been deployed to inspect the extracted teeth to make sure they had the following inclusion criteria; fully formed roots with mature apices, and there was no sign of resorption or abnormal defect (fractured or cracked root). At the same time, the exclusion criteria include the presence of any calcified root canals, previous root canal treatment, internal and external resorption, and not fully formed root apex [16]. The investigators collected the extracted maxillary first premolar teeth from the Oral Surgery Department, and several private dental clinics.

One hundred forty-two left and right first maxillary premolars, which had been previously extracted because of caries, trauma, periodontal disease, or orthodontic reasons, were selected. The participants’ age ranged from 20 to 45 years old, while the gender of the individuals was not included in the records. However, all teeth were identified as maxillary first premolars at the extraction time by the operator.

They were washed after extraction and stored in a 10% formalin solution until the collection was complete [17]. Each tooth was cleaned of adherent soft tissues, bone fragments, and calculus by scaling and polishing. Tap water was used to irrigate and wash the teeth, then dried using a 95% ethanol solution.

Inclusion criteria include fully formed roots with mature apices, and there is no sign of resorption or abnormal defect (fractured or cracked root). At the same time, the exclusion criteria include the presence of any calcified root canals, previous root canal treatment, internal and external resorption, and not fully formed root apex.

Two experienced investigators visually inspected the root morphology and recorded the findings. The teeth were classified according to the forms and number of roots a follows [18]:

Class a: Single root, single tipped.
Class b: Single root, the small double apex can be seen or felt.
Class c: Single root, bifid apex (bifurcation less than one-fourth to one-third of the buccal root length).
Class d: Two roots (bifurcation exceeds one-fourth to one-third of the buccal root length).
Class e: Three roots (two buccal and one palatal).

After visual inspection, the teeth were radiographed in mesiodistal and distomesial plains, and the direction of the beam was set at 40 degrees horizontal angulation to evaluate the root canal morphology using digital radiography (TOSHIBA D-0712, Japan) [19]. Exposure parameters of the x-ray were set at 65 kVp, 7.5 mA, and an exposure time of 0.20 s. The distance between the buccal surface and the focal spot was 20 inches. Digital images were taken by a direct system using a CCD receptor of 23 × 14 × 4 mm size and displayed in an LG 14-inch monitor with 1600 × 1200 resolution. The images were prepared with Kodak software and saved in RVG format [20].

The long axis of the root is placed parallel and near the surface of the X-ray sensor (EzSensor Classic, Vatech, Korea), type HD, size 1.5. a standardized parallel technique Periapical radiographs were obtained for all the teeth using a film holder (XCP; Rinn, Elgin, IL). A high-frequency oral X-ray machine was used with an exposure time of 0.367 s (60 kV, 4 mA). Two endodontists evaluated all the teeth and their corresponding radiographs utilizing digital radiography, and they were equipped to expand the computerized photo on the computer and extract precise and accurate details. They examined each specimen several times until reaching a consensus concurrently.

Examination of the root canal system of the teeth was based on Vertucci's classification of root canal morphology [21]:

Type I A single main canal is present from the pulp chamber to the root apex.
Type II Two separate canals leave the pulp chamber and merge to one canal to the apex.
Type III One canal leaves the pulp chamber and divides into two smaller canals that later merge to exit one canal.
Type IV Two separate and completely distinct canals run from the pulp chamber to the root apex.
Type V A single canal exiting the pulp chamber, which divides into two canals with separate apical foramina.
Type VI Two separate canals join at the middle of the root to form one canal, which extends till the apex, just short of the apex, and again divides into two.
Type VII The canal starts as a single until the middle third of the root, then divides into two separate canals that rejoin after some distance and then, near the apex, divides into two again.
Type VIII The pulp chamber near the coronal portion divides into three separate canals extending till the apex.

According to Vertucci's classification, all teeth were examined using digital radiography. Then, the teeth specimens were sliced mesiodistally into slices to verify the internal root canal anatomy, using a low-speed diamond saw (200 rpm) with a loading of 50 g (Isomet, Buehler Ltd., USA). Finally, for more accessible and more accurate visualization of the canal details, the canal configuration, the pulp chamber, and roots canals were highlighted (marked) with a fine tip marker permanent orange color (Staedtler, Lumocolor, Germany).

### Statistical analysis

The actual data is being rearranged and transported to SPSS Statistics for Windows, version 24.0 (Armonk, NY: IBM) software. The sample size was calculated with the Sealed Envelope software.

## Results

### Morphology and number of roots

**Fig. 1:**
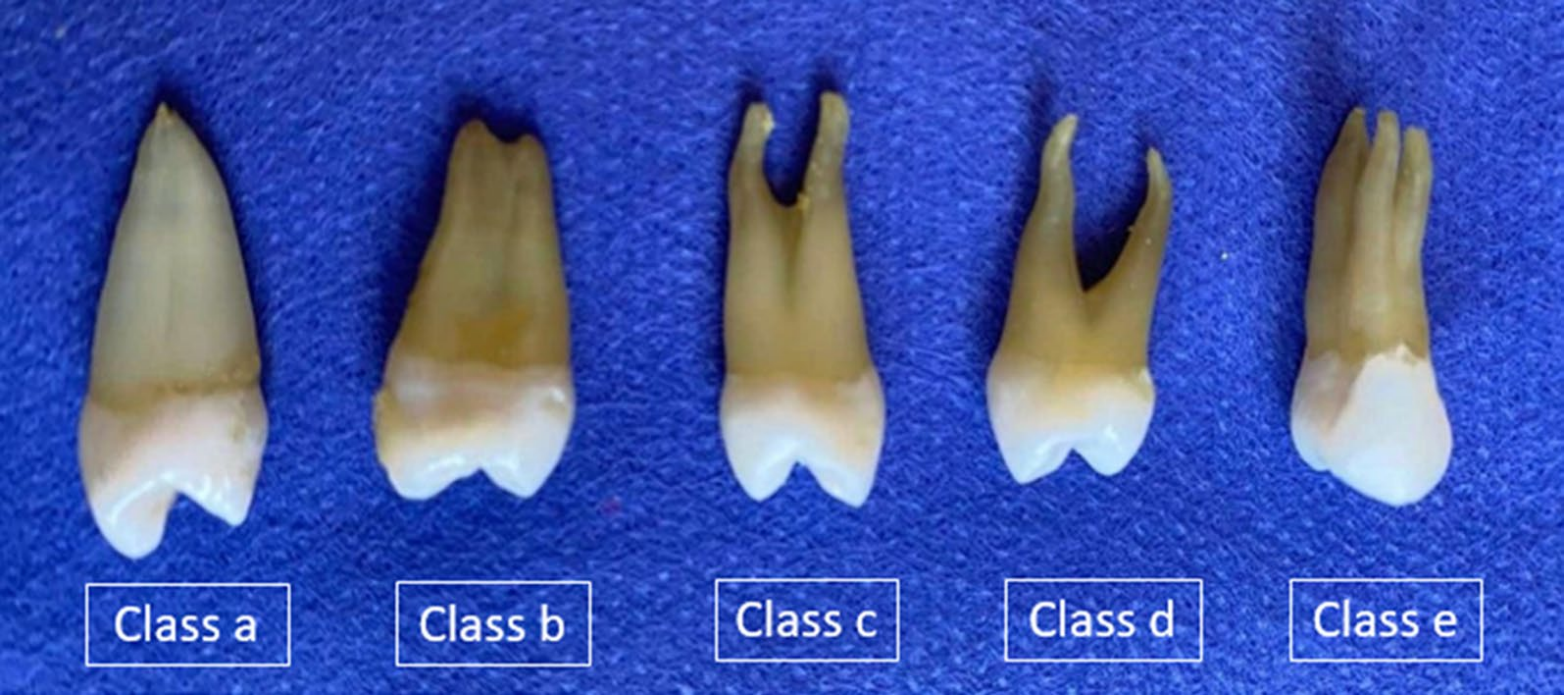
Diagrammatic representation of the morphology, number, and different classes of maxillary premolar root

From the 142 maxillary first premolars studied, 42 teeth (29.58%) had one root, while two teeth (1.41%) were single-tipped root apex and the rest; 40 teeth (28.17%) had double-tipped root apex (Table 1). Thus, out of 97 teeth (68.31%) double-rooted premolars, 34 teeth (23.94%) had two fused roots (they exhibited bifurcation in the apical third) and 63 teeth (44.37%) had two separated roots (the furcation of the roots was clearly defined and consistently started at a level about half of the lengths of the two roots). Three premolars (2.11%) of the tested samples had three separated roots (Table 1, Fig. 1).

**Table 1:** The frequency distribution and percentage of the root numbers of maxillary permanent first premolars in a sample of the Sulaimani population

No. of root	No. of tooth	Types of the root morphology	No. of tooth	(n) %	
Single-rooted	42	Single-tipped root apex (a)	2	1.40	29.57
		Double-tipped root apex (b)	40	28.17	
Double-rooted	97	Fused two roots (c)	34	23.94	68.31
		Separate two roots (d)	63	44.37	
Three-rooted	3	Three roots (e)	3	2.12	
Total			142	100%

### Number and configuration of root canals

**Fig. 2:**
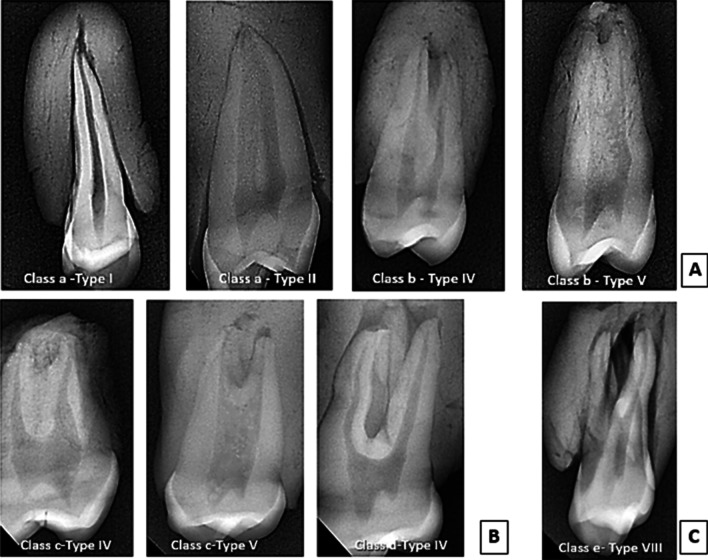
Representative radiographic images show the canal configuration patterns of maxillary first premolar; **a** single-rooted specimens showing different root canal configurations, **b** double-rooted specimens showing different canal configurations, **c** three-rooted specimen with type VIII canal configuration

**Fig. 3:**
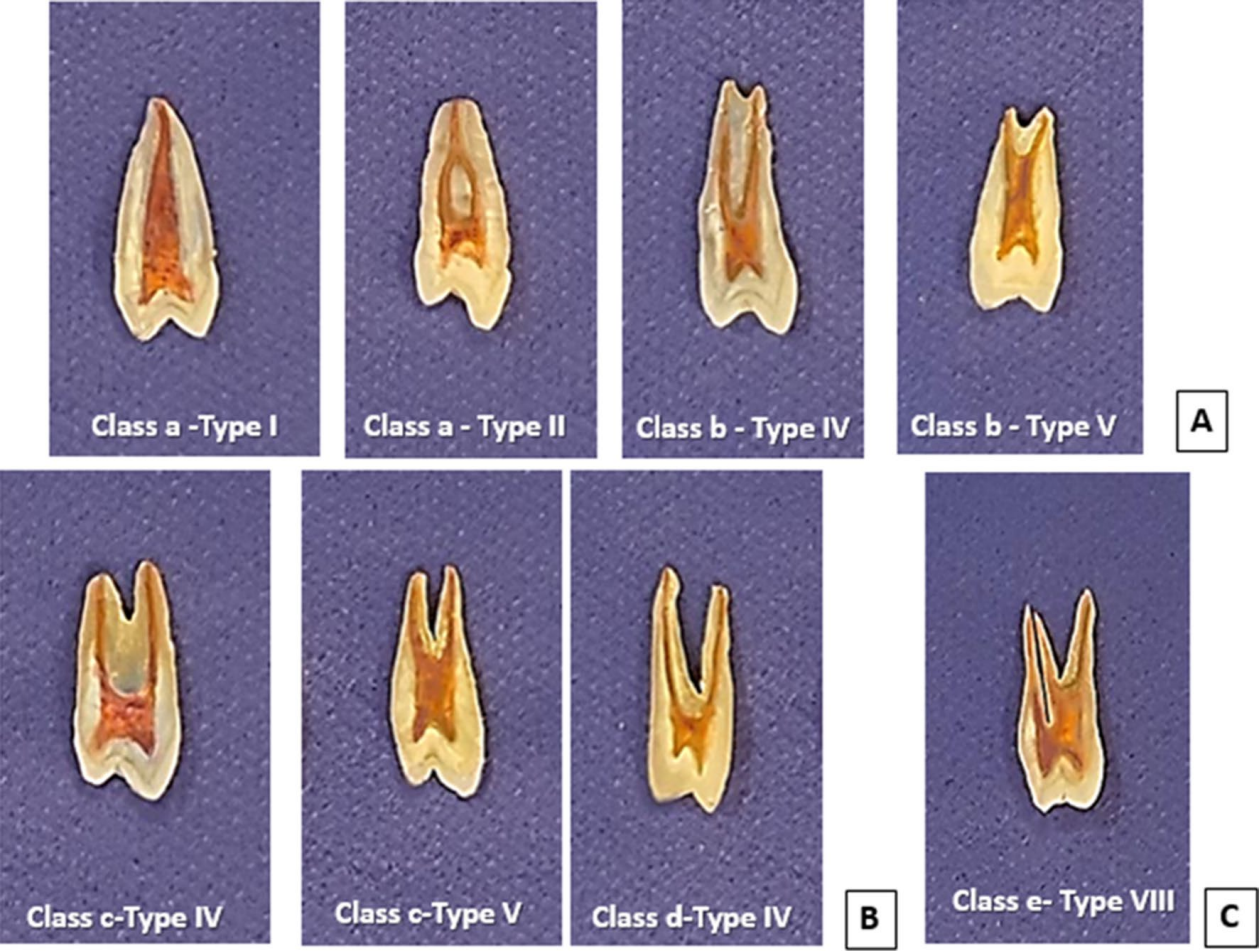
Sliced teeth specimens with pulp chamber and root canals highlighted with a fine tip marker showing the types of canal configuration of the maxillary first premolar; **a** single-rooted specimens showing different root canal configurations, **b** double-rooted specimens showing different canal configurations, **c** three-rooted specimen with type VIII canal configuration

Single-rooted premolars demonstrated a wide variation of root canal configurations; in single-tipped apex, only one tooth (0.70%) had type I configuration, and one tooth (0.70%) had type II configuration. Whereas in double-tipped root apex, 13 teeth (9.16%) had type IV configuration, and 27 teeth (19.01%) had type V configuration, as shown in (Table 2 and Figs. 2A, 3A).

**Table 2:** The frequency and percentage of canal system type according to Vertucci’s classification in maxillary first permanent premolars in a sample of Sulaimani population

Canal configurations	Single-rooted (%)		Double-rooted (%)		Three-rooted (e)	Total (%)
	Single-tipped root apex (a)	Double-tipped root apex (b)	Fused two roots (c)	Separate two roots (d)		
Type I	1 (0.70)	0	0	0	0	1 (0.70)
Type II	1 (0.70)	0	0	0	0	1 (0.70)
Type III	0	0	0	0	0	0
Type IV	0	13 (9.16%)	24 (16.90)	63 (44.37)	0	100 (70.43)
Type V	0	27 (19.01%)	10 (7.04)	0	0	37 (26.05)
Type VI	0	0	0	0	0	0
Type VII	0	0	0	0	0	0
Type VIII	0	0	0	0	3 (2.12)	3 (2.12)
Total (%)	2 (1.40)	40 (28.17)	34 (23.94)	63 (44.37)	3 (2.12)	142 (100)

On the other hand, of the specimens that had double roots with fused two roots patterns, 24 teeth (16.90%) had type IV configuration, and only ten teeth (7.04%) had type V root canal configuration. While in double roots with separated two roots, all the 63 teeth (44.37%) had type IV root canal configuration as shown in (Table 2 and Figs. 2B, 3B). Finally, all the three teeth (2.12%) that had three roots had type VIII root canal configuration (Table 2 and Figs. 2C, 3C). The frequency of root canals found in maxillary first premolar teeth observed in several studies, alongside this study's results, is summarized in Table 3.

**Table 3:** Percentages of root canal configurations found in maxillary first premolars in the present and previous studies

References	Year	Population	Teeth no	Method	Vertucci’s root canal Configurations (%)							
					Type I	Type II	Type III	Type IV	Type V	Type VI	Type VII	Type VIII
Vertucci and Gegauff [12]	1979	North America	400	Clearing	8	18	–	62	7	–	–	5
Kartal et al. [30]	1998	Turkey	300	Clearing	8.7	1	–	71.3	14.7	2.3	0.3	1.3
Chaparro et al. [32]	1999	Andalusia	150	Radiograph	1.3	37.3	–	58	–	–	–	3.3
Sert and Bayirli [34]	2004	Turkey	221	OP & CDR	10.5	12.5	5.5	61.5	3.5	1	–	3
Lipski et al. [35]	2005	Poland	142	Radiograph	2.1	6.3	–	82.4	–	–	–	9.2
Peiris [43]	2008	Sri Lanka	153	Clearing	1.3	16.3	2	64	5.9	5.9	0.7	–
		Japan	81		4.9	29.6	2.5	45.7	2.5	8.6	–	–
Atieh et al. [8]	2008	Saudia	246	Radiograph	8.9	26.8	–	63	–	–	–	1.2
Awawdeh et al. [27]	2008	Jordan	600	Clearing	3.3	10.2	0.3	79.7	2	2.3	–	1.5
Rwenyonyi et al. [36]	2011	Ugandan	202	M-clearing	13.0	29.6	1.9	71.6	1.9	1.9	3.7	3.4
Tian et al. [44]	2012	Chinese	241	CBCT		23		51				1
Gupta et al. [19]	2015	North India	250	Clearing	23.2	14.8	13.6	33.2	6.8	2	4	0.4
Senan et al. [42]	2018	Yemen	250	Clearing	13.2	4.4	8.0	55.6	5.6	1.6	3.6	0.8
ALqedairi et al. [31]	2018	Saudi	707	CBCT	10.8	8.4	1.8	70.6	3.9	2.1	0.3	2.1
Liu et al. [41]	2019	Chinese Adolescent	324	Micro-CT	25	8.33	2.78	52.78	5.56	2.78	1.54	1.23
Maghfuri et al. [29]	2019	Saudi	100	CBCT	0	7	0	75	13	2	0	3
Wu et al. [40]	2020	Shandong Chinese	1268	CBCT	10.4	24.2	0.39	58.6	4.9	0.87	0	0.39
Asheghi et al. [47]	2020	Iran	462	CBCT	8.87	15.15	0.86	71.64	1.30	0.22	0	1.95
Kfir et al. [20]	2020	Israel	400	CBCT	2	17	0.5	74	0.5	6	–	–
Al‑Zubaidi et al. [28]	2021	Saudia Arabia	500	CBCT	5.2	32.8	0.6	57.8	2.0	–	–	1.6
Present study	2021	Sulaimani	142	DR	0.70	0.70	–	70.43	26.05	–	–	2.12

## Discussion

Accurate knowledge of the root canal morphology and its anatomical variations is mandatory for successful root canal treatment [22]. The maxillary first premolars can be considered one of the most challenging teeth to treat endodontically due to their variation in the roots, canal configuration, direction and longitudinal depressions, and various pulp cavity configurations [23]. Therefore, maxillary first premolars from a sample of the Iraqi subpopulation were chosen for this study.

Different root canal morphology varies significantly among various populations, and hence there is a difference in the study results across the globe [24]. Multiple methods have been used in literature to investigate root canal anatomy [25–28], including radiographic examination [19, 29–31]. A variable number of roots were identified as single root, two roots, and three were found in maxillary first premolars; on the other hand, there may be one to three canals per root [32, 33]. In addition, several studies evaluated the morphology of maxillary first premolar teeth, leading to the discovery that significant variations related to ancestry or geographic origins have been noted [27, 34–36].

The present ex-vivo study evaluated 142 extracted first maxillary premolars using digital radiography and sectioning methods. A high percentage of root morphology was two roots, followed by one root and three roots. Similar results were found in previous studies conducted in Saudi Arabian subpopulations, Jordanian population, and Turkish populations, which also observed a higher prevalence of two roots followed by one root than three roots [19, 28, 37–40].

Some studies showed results that were near to our findings regarding the prevalence of the number of roots with a slight variation in the percentage of the number of roots, like the studies that have been conducted in Saudi, North America, Andalusia, Singapore, Turkey, Poland, Ugandan, San Sebastian, southern India, and Australia population [5, 17, 21, 41–48]..On the contrary, studies in the Shandong Chinese population showed a higher prevalence of one-rooted than two-rooted maxillary first premolar [6, 49]. Other studies also contradict our study, which has been done in north India, Yemen, Seri Lankan, Japan, China, Brazil, and the Iranian population [27, 35, 36].

Regarding the description of one root, whether they were single-tipped root apex (class a) or double-tipped root apex (class b). The double-tipped root apex was more prevalent than the single-tipped root apex in the present study. On the contrary, a survey by Senan et al*.* [35] and Peirisa [50] showed a higher incidence of single-tipped root apex than a double-tipped root apex.

On the other hand, the root patterns were also analyzed in this study. The result showed a higher incidence of separated two-roots than fused two-roots. Unfortunately, many studies did not mention the details of the two roots, whether they were fused or separate [5, 27, 36, 38, 47, 48], but a survey by Rwenyonyi et al. [46] showed a close result with our study. Meanwhile, a study by Loh et al*.* [43], Gupta et al*.* [27], and Senan et al. [35] showed that the fused two-roots had a higher incidence than the separated two-roots in Singapore, north India, and Yemen population, respectively.

In the present study, the lowest incidence of three-rooted maxillary first premolar was observed compared to single-rooted and two-rooted. This result agrees with Rwenyonyi et al*.* [46], Tian et al*.* [51], and Pecora et al. [52]. In contrast. Loh et al. [43] and Walker [53] reported no three roots in their study samples. The differences among the results of these studies could be attributed to variations in examination methods, classification systems, sample sizes, and ethnic backgrounds of the population [27].

In the present study, the Vertucci classification was selected as a base for the exploration of root canal configurations since it is the most commonly used classification, and the maxillary first premolar is the tooth that was showing all the eight types of canal configurations, in addition, it was utilized in the current study for easy comparison with the results of other investigations. The type IV root-canal morphology was the most common type seen in the present study and the similar studies reviewed in the literature but with variations in the percentage and frequency-specific for different kinds of canal configurations [36–40], except in the teeth with the three roots which showed only type VIII. This result agrees with Wu et al*.* [49], Liu et al. [6], Senan et al. [35], and Asheghi et al. [36], who found type VIII in all three rooted maxillary first premolar.

Due to the number of tested teeth leading to the relatively small size included in the present study, the root and canal morphology patterns should be considered since they could not represent the larger population of Iraq. However, further studies are recommended to explore the tooth morphology in the Iraqi population using a more significant number of samples and more advanced techniques, such as dissecting light microscope, CBCT, and computed tomography, to study the root canal morphology. The sample size and conventional approach used to determine the precise root canal morphology are prevalent limitations of this research. Clinically, it is crucial to identify the root-canal anatomy prevalent in a population to reduce errors during endodontic treatment.

## Conclusions

The present study showed a high incidence of two-rooted with separate roots and predominantly have Type IV Vertucci's configuration. Nevertheless, the present study results further confirm the importance of a thorough knowledge of root canal morphology for each population and the need for a careful exploration and radiographic examination of these teeth before endodontic therapy.

## Data Availability

The datasets generated and/or analyzed during the current study are not publicly available due to privacy and ethical concerns but are available from the corresponding author on reasonable request.
